# Supplementary material for: COVID-19 and self-reported health of the Norwegian adult general population: A longitudinal study 3 months before and 9 months into the pandemic
Source: PLoS One. 2024 Oct 24;19(10):e0312201. doi: 10.1371/journal.pone.0312201 (PMC11500952; doi:10.1371/journal.pone.0312201)
Supplement: S1 Table — (DOCX) [file pone.0312201.s001.docx]

**S1 Table Mean (SD) change in EQ-5D and PROMIS-29 scores for socioeconomic groups and self-reported health problems**

|  |  |  |  | PROMIS-29 domains^c^ | | | | | | | |
| --- | --- | --- | --- | --- | --- | --- | --- | --- | --- | --- | --- |
|  | N | EQ-5D Index^a^ | EQ VAS^b^ | Anxiety | Depression | Fatigue | Pain intensity^d^ | Pain interference | Physical function | Sleep disturbance | Social participation |
| All | 2417 | -0.001 (0.123) | -1.43 (12.87) | 0.48 (6.52) | 0.49 (6.19) | -0.00 (7.39) | 0.14 (1.51) | 0.41 (5.95) | -0.26 (4.50) | 0.09 (6.65) | -1.01 (7.26) |
| Gender |  |  |  |  |  |  |  |  |  |  |  |
| Female | 1312 | -0.004 (0.126) | -1.63 (13.17) | 0.68 (6.79) | 0.61 (6.38) | 0.27 (7.82) | 0.14 (1.54) | 0.56 (6.10) | -0.24 (4.28) | 0.21 (6.99) | -1.18 (7.20) |
| Male | 1097 | 0.002 (0.118) | -1.24 (12.54) | 0.25 (6.17) | 0.35 (5.95) | -0.32 (6.85) | 0.15 (1.48) | 0.22 (5.76) | -0.33 (4.28) | -0.07 (6.20) | -0.84 (7.32) |
| Age years |  |  |  |  |  |  |  |  |  |  |  |
| 18-29 | 429 | -0.004 (0.137) | -1.07 (14.51) | 0.82 (7.68) | 0.88 (7.12) | 0.09 (8.23) | 0.09 (1.50) | 0.13 (6.02) | -0.05 (4.07) | 0.47 (8.20) | -0.72 (7.49) |
| 30-59 | 897 | 0.005 (0.118) | -0.97 (12.47) | 0.26 (6.22) | 0.10 (6.13) | -0.01 (7.67) | 0.07 (1.48) | 0.31 (5.99) | -0.04 (4.17) | -0.03 (6.72) | -0.72 (7.55) |
| 60-79 | 873 | -0.002 (0.117) | -2.07 (12.17) | 0.30 (6.16) | 0.59 (5.67) | -0.17 (6.74) | 0.21 (1.52) | 0.64 (5.74) | -0.30 (4.83) | 0.03 (5.89) | -1.29 (6.66) |
| 80+ | 199 | -0.021 (0.133) | -1.50 (13.59) | 1.52 (6.57) | 0.98 (6.39) | 0.54 (6.84) | 0.35 (1.65) | 0.49 (6.57) | -1.62 (5.10)* | 0.44 (5.77) | -1.69 (7.57) |
| Education level |  |  |  |  |  |  |  |  |  |  |  |
| School | 1112 | 0.004 (0.128) | -1.34 (14.11) | 0.31 (6.75) | 0.25 (6.34) | 0.11 (7.42) | 0.12 (1.63) | 0.32 (6.17) | -0.25 (4.77) | -0.28 ( 6.74) | -0.85 (7.25) |
| Higher | 1296 | -0.006 (0.118) | -1.50 (11.73) | 0.61 (6.31) | 0.70 (6.04) | 0.05 (7.38) | 0.16 (1.40) | 0.46 (5.77) | -0.28 (4.24) | 0.41 (6.57) | -1.17 (7.28) |
| Cohabiting | 1658 | -0.003 (0.118) | -1.76 (12.04) | 0.60 (6.45) | 0.48 (6.14) | -0.06 (7.20) | 0.17 (1.51) | 0.59 (5.99) | -0.30 (4.43) | 0.13 (6.57) | -1.07 (7.23) |
| Born outside Norway | 177 | 0.001 (0.128) | -0.75 (14.11) | 0.87 (6.78) | 0.41 (5.59) | -0.41 (8.34) | 0.22 (1.64) | 0.50 (6.86) | 0.35 (3.71) | 0.67 (6.88) | -1.00 (8.18) |
| SCQ comorbidity |  |  |  |  |  |  |  |  |  |  |  |
| Heart disease | 247 | -0.003 (0.127) | -1.40 (11.97) | -0.83 (6.33)* | 0.14 (5.95) | -0.21 (7.13) | 0.14 (1.62) | 0.02 (5.61) | -0.85 (5.34) | -0.17 (5.46) | -0.78 (7.26) |
| High blood pressure | 508 | -0.006 (0.131) | -1.99 (12.94) | 0.98 (6.53) | 1.13 (6.03)* | 0.22 (6.91) | 0.26 (1.58) | 0.57 (6.10) | -0.41 (4.89) | 0.34 (6.02) | -1.50 (7.11) |
| Lung disease | 148 | 0.016 (0.131) | -2.10 (14.57) | -0.46 (6.78) | -0.29 (6.27) | -0.32 (7.05) | 0.23 (1.56) | 0.41 (6.36) | -0.77 (5.10) | 0.07 (6.68) | -1.00 (6.32) |
| Diabetes | 132 | -0.000 (0.146) | -1.12 (15.83) | -0.26 (6.59) | 0.37 (6.48) | -0.08 (7.78) | 0.40 (1.87) | 1.04 (7.24) | -0.37 (5.00) | 0.61 (5.97) | -0.57 (7.01) |
| Stomach ulcer/disease | 172 | -0.003 (0.137) | -2.55 (13.26) | -0.06 (6.66) | -0.13 (6.74) | -0.21 (7.36) | 0.15 (1.73) | 0.41 (7.06) | -0.70 (5.57) | 0.50 (6.70) | -1.45 (7.04) |
| Liver disease | 24 | 0.011 (0.145) | 0.57 (21.13) | -1.54 (7.32) | -2.87 (7.28) | -5.13 (8.95)* | 0.13 (1.83) | 0.37 (6.73) | 0.10 (4.08) | 0.89 (8.32) | 0.53 (5.20) |
| Kidney disease | 35 | 0.001 (0.154) | -3.33 (15.27) | -0.28 (7.19) | 1.23 (4.93) | -1.06 (7.18) | 0.06 (1.86) | 0.17 (7.60) | -0.84 (5.78) | 0.37 (6.64) | -1.61 (6.53) |
| Anemia/blood disease | 31 | 0.010 (0.127) | -1.03 (12.16) | -0.75 (6.42) | -1.52 (8.39) | -1.85 (6.98) | 0.10 (1.14) | 1.60 (5.86) | -1.20 (4.37) | -2.14 (7.03) | -1.44 (6.12) |
| Cancer | 84 | 0.007 (0.106) | -2.20 (12.94) | -1.59 (6.51)* | -0.43 (5.94) | -0.55 (8.06) | 0.30 (1.67) | 0.96 (6.34) | -0.58 (4.55) | -0.12 (5.09) | -1.02 (5.99) |
| Depression | 259 | 0.022 (0.151)* | -0.87 (15.91) | -1.73 (7.12)* | -1.65 (6.70)* | -1.26 (7.91)* | 0.02 (1.79) | 0.08 (6.32) | -0.57 (5.78) | -0.35 (6.78) | 0.28 (7.29)* |
| Arthritis | 604 | 0.000 (0.132) | -2.05 (13.25) | 0.02 (6.46) | 0.53 (6.19) | -0.14 (6.74) | 0.22 (1.60) | 0.26 (5.74) | -0.39 (5.29) | 0.05 (6.16) | -0.83 (7.02) |
| Back pain | 738 | 0.016 (0.136)* | -1.53 (13.15) | -0.32 (6.54)* | 0.19 (6.30) | -0.63 (7.38)* | -0.01 (1.69)* | -0.27 (6.09)* | -0.07 (5.25) | -0.01 (6.78) | -0.50 (7.18) |
| 0 | 602 | -0.015 (0.110)* | -1.50 (12.88) | 1.05 (6.24)* | 0.67 (5.86) | 0.49 (7.23) | 0.22 (1.21) | 0.92 (4.97)* | -0.21 (2.80) | 0.43 (6.81) | -1.47 (7.30) |
| 1 | 619 | -0.005 (0.12) | -1.98 (12.51) | 0.65 (6.34) | 0.58 (6.08) | 0.38 (7.63) | 0.18 (1.60) | 0.45 (6.51) | -0.08 (4.17) | 0.33 (6.80) | -1.21 (7.73) |
| 2 | 520 | 0.007 (0.119) | -0.77 (11.83) | 0.45 (6.84) | 0.48 (6.43) | -0.32 (7.42) | 0.02 (1.56) | 0.35 (6.41) | -0.35 (5.04) | -0.25 (6.25) | -0.65 (7.02) |
| 3+ | 581 | 0.010 (0.141) | -1.54 (14.11) | -0.41 (6.59)* | 0.16 (6.41) | -0.62 (7.39) | 0.14 (1.69) | -0.13 (5.98) | -0.43 (5.57) | -0.11 (6.67) | -0.60 (7.16) |

^a^ EQ-5D-5L index scores range from -0.57 to 1 where 1 is the best possible health state.

^b^ EQ VAS scores range from 0-100 where 100 is the best possible health state. Positive change scores represent improvement.

^c^Domains are T-scores where a score of 50 is the average for the US general population with a standard deviation of 10. Higher scores for domains and items represent more of a domain, for example, higher levels of physical functioning or anxiety. Changes scores for anxiety, depression, fatigue, pain interference/intensity, sleep disturbance: negative values represent improvement. Change scores for physical function and social participation: positive values represent improvement.

^d^Numerical rating scale from 0-10; 0 is lowest and 10 the greatest pain intensity.

Asterisks denote statistically significant differences in comparisons with the remainder of respondents (P<0.01).
