# Supplementary material for: COVID-19 and self-reported health of the Norwegian adult general population: A longitudinal study 3 months before and 9 months into the pandemic
Source: PLoS One. 2024 Oct 24;19(10):e0312201. doi: 10.1371/journal.pone.0312201 (PMC11500952; doi:10.1371/journal.pone.0312201)
Supplement: Attachment — Submitted filename: 4. PONE-D-24-18493.docx [file pone.0312201.s002.docx]

**Review report** on paper titled “COVID-19 and self-reported health of the Norwegian adult general population: a longitudinal study 3 months before and 9 months into the pandemic”

**Comments**

Title

- No comments.

**Abstract**

- What is the motivation behind investigating this specific area?
- Which sampling method was utilized?
- What are the key conclusions drawn?
- Can you provide some keywords that encapsulate the essence of the study?

**Introduction**

- The duration of the Covid-19 pandemic in Norway and its impact on the Norwegian adult population are important topics to explore. To effectively engage readers, it is vital to present compelling evidence that showcases the duration of the pandemic in the area. Does it really spanned over duration of 9-months?
- Rephrase “In November 2020, 3200 respondents to a representative general population survey in Norway in December 2019 received a follow-up postal questionnaire including the EQ-5D-5L, PROMIS-29 instruments, and questions about respondents having or having had COVID-19”

**Materials and methods**

- Statistical Power for sample size?
- Is there a difference in the content of the questionnaires used in baseline and follow-up studies? Clarity is needed regarding whether the questionnaire content should remain consistent or be varied.
- What particular strategies did the authors utilize to control the influence of variables that could impact the minimal important change (MIC) and age-related changes? Or how could you specifically relate health outcomes with COVID-19 only?
- Were there any specific methods employed by the authors to follow the participants throughout the study? Or to overcome the challenges due to participants dropping out over time, which can affect the validity of the results.
- How could authors overcome or capture short-term health outcomes?

**Report**

- Nice to see educational status of the participants in report
- No further comment

**Discussion**

- The discussion fails to provide an explanation for the potential factors influencing the results; rather, it simply compares them to previous findings, emphasizing the need for a revision.

**Strengths and limitations**

- Too long

**Conclusion:**

- No comment

**Recommendation**

- Needed.

**References:**

- No comments.
