# Supplementary material for: COVID-19 and self-reported health of the Norwegian adult general population: A longitudinal study 3 months before and 9 months into the pandemic
Source: PLoS One. 2024 Oct 24;19(10):e0312201. doi: 10.1371/journal.pone.0312201 (PMC11500952; doi:10.1371/journal.pone.0312201)
Supplement: Attachment — Submitted filename: Response_to_Reviewers_PONE_D_24_18493.docx [file pone.0312201.s003.docx]

**Review report** on paper titled “COVID-19 and self-reported health of the Norwegian adult general population: a longitudinal study 3 months before and 9 months into the pandemic”

**Editor’s Comments**

**Abstract**

1. What is the motivation behind investigating this specific area?

The first sentence of the abstract now explicitly states that the study aim was to assess the impact of the pandemic on broader aspects of health in the general population (Abstract, lines 17-18).

2. Which sampling method was utilized?

We have now stated that sampling for the baseline study followed the results of similar Norwegian surveys designed for the collection of general population norms. We have given the sample size and stated that they were randomly selected (Abstract, lines 20-21).

3. What are the key conclusions drawn?

We have included a final sentence in the abstract which concludes that based on the study findings, there was no evidence for a decline in important aspects of general population health (Abstract, lines 39-41).

4. Can you provide some keywords that encapsulate the essence of the study?

Keywords now include COVID-19, EQ-5D-5L, general population, PROMIS-29, survey (line 43).

**Introduction**

5. The duration of the Covid-19 pandemic in Norway and its impact on the Norwegian adult population are important topics to explore. To effectively engage readers, it is vital to present compelling evidence that showcases the duration of the pandemic in the area. Does it really spanned over duration of 9-months?

We have now included information on the duration of the first and second waves of the pandemic in Norway which are most relevant to the article. We have also included information on the government response including lockdown measures, together with references (page 5, lines 93-96; additional references 12-14). We have also stated that the follow-up survey coincided with the second wave (page 6, lines 98-99).

6. Rephrase “In November 2020, 3200 respondents to a representative general population survey in Norway in December 2019 received a follow-up postal questionnaire including the EQ-5D-5L, PROMIS-29 instruments, and questions about respondents having or having had COVID-19”

This has been rephrased following further information being given about sampling in response to the point above (Abstract, lines 21-24).

**Materials and methods**

7. Statistical Power for sample size?

We have now stated the sample size for the published baseline component of the study was based on a review of existing Norwegian studies that had similar aims. The reference used is already included in the reference list (page 6 lines 104-106).

8. Is there a difference in the content of the questionnaires used in baseline and follow-up studies? Clarity is needed regarding whether the questionnaire content should remain consistent or be varied.

We have explicitly stated that the EQ-5D-5L, PROMIS-29 and SCQ were included at baseline. We have also stated that the SCQ was not included and that the COVID-19 related questions were included at follow-up (pages 6-7, lines 123-127).

9. What particular strategies did the authors utilize to control the influence of variables that could impact the minimal important change (MIC) and age-related changes? Or how could you specifically relate health outcomes with COVID-19 only?

Given that this was a general population cohort study, it was only possible to control for the variables collected by means of the two questionnaires including baseline health and respondent characteristics, including age, as independent variables in the multivariate regression analyses (page 8, lines 165-167; Tables 3-4).

The minimal important change (MIC) is the is the smallest change above which individuals themselves perceive as important (reference 24). This follows from the measurement properties of the instruments as well as the study population, although there is no universal method for deriving MICs. This study was not designed to determine the MIC, as we had no external anchors to determine whether changes were meaningful or not. We therefore used available MIC from other studies, as reported in the Methods (references 19-24). In the Discussion, we have added that we compare with previously reported MIC estimates for these instruments (page 14, lines 224-225).

10. Were there any specific methods employed by the authors to follow the participants throughout the study? Or to overcome the challenges due to participants dropping out over time, which can affect the validity of the results.

Reminders were not used at baseline or follow-up but may have helped increase the response rates slightly. We used a lottery incentive at baseline and continued with this at follow-up. We have extended the strengths and limitations section of the discussion accordingly (page 17, lines 300-301).

11. How could authors overcome or capture short-term health outcomes?

We might have assessed short-term health outcomes by means of an additional questionnaire during the first wave of the pandemic. We have now stated this in the discussion (page 16, lines 291-292).

**Discussion**

12. The discussion fails to provide an explanation for the potential factors influencing the results; rather, it simply compares them to previous findings, emphasizing the need for a revision.

We have added a new paragraph to the discussion which gives and explanation of factors which may have influenced the results. The findings are in line with a report commissioned by the Norwegian government. We have included a reference [14] along with consideration to when vaccination against COVID-19 started in Norway (page 14, lines 229-240) with an additional reference [27].

**Strengths and limitations**

13. Too long

This section was shortened without loss of specific study strengths and limitations. We have shortened the strengths component relating to aspects of data collection (page 16, lines 274-288). We have shortened the limitations component relating to generic and specific patient-reported outcome measures (page 17, lines 302-304).

**Recommendation**

14. Needed.

At the close of the conclusions, we have stated that interventions are needed for potentially vulnerable groups (page 17, lines 315-316).
